# Pellagra—Its Cause, Prevention and Cure

**Published:** 1916-08

**Authors:** E. M. Perdue

**Affiliations:** Professor of Tropical Medicine in Eclectic Medical University, Kansas City, Mo.


### Pellagra—Its Cause, Prevention and Cure.

BY E. M. PERDUE, A. M., M. D., D. P. H., PROFESSOR OF TROPICAL MEDICINE IN ECLECTIC MEDICAL UNIVERSITY, KANSAS CITY, MO.

Pellagra is a subject of very great importance in certain regions in the southern part of the United States for the following reasons:

1. The disease or condition is increasing the number of its victims and extending its boundaries.

2. The number of cases and the mortality have become an economic problem in the endemic area.

3. Its specific cause, prevention and treatment were only discovered as recently as 1913.

4. There is a concerted effort among certain medical and research interests in America to discredit the proof of the cause of pellagra and thereby hinder its eradication.

5. The public and the profession, especially in the South, are entitled to know the cause and cure of pellagra, and also to know of the effort to mislead them and obscure the issue.

The specific cause of pellagra was first authoritatively stated in May, 1913. The statement came from two of the foremost scientists of the Institute of Experimental Hygiene of the University of Rome. They had been concerned in a systematic research since 1909. Their conclusions are based upon findings in a research conducted according to the most scientific criteria and conducted with the laboratory animals and confirmed by the successful treatment of pellagrous patients. The methods of prevention and treatment suggested by these illustrious investigators have been adopted with success by the Italian governmental agencies. Their research has been published in English. It is known to the great research laboratories in America. But it came from Italy and not from Germany, and was not accepted. This failure of acceptance was not because the research was not final and conclusive, but because it did not fit certain pet hypotheses and did not come from favored sources. Since its publication many so-called researches have been devised for the purpose of discrediting the Italian findings. One of these emanates from the United States Public Health Service and enjoys the prestige and frank of the government. Another very recent attack comes from Tennessee. Both of these attacks evidence the poverty of scientific acumen which is such a disappointing characteristic of American research. Scientific facts, clinical proof, endemic and epidemic conditions are all ignored in order to discredit truth and bolster up a pet hypothesis. The humanities, the good of the suffering, the prevention of disease, are all disregarded in favor of hypothesis.

According to Professors Alessandrini and Scala of the University of Rome, pellagra is a chronic acid intoxication caused by colloidal silica in drinking water derived from clay soils deficient in carbonate alkalies. This is the cause of pellagra in Italy, in Spain, Portugal, the Tyrol, Roumania and Bessarabia. The soils of these countries are largely clays derived from the weathering and disintegration of the igneous and crystalline rocks of their principal mountain chains. These soils have lost their small percentage of original alkali through the leaching and erosion occasioned by the culture of a large and fixed agricultural population. The soils lack the essential alkaline base furnished by the limestones of the great inland regions of the continent. These regions of acid soil were originally covered by pine timber. The regions of endemic pellagra in Europe coincide with the geological distribution of clay soils and the geographical distribution of pine timber. Alkaline prairies are practically immune.

Since these facts and many others of similar import are established for Europe, scientific candor and fairness, to say nothing of humanitarian motives, would dictate their application to like conditions in America. A most cursory review of the epidemiology, and demography of pellagra in this country at once correlates it with the geological conditions prevailing in like areas in Europe and absolutely essential to its prevalence in any place in the world. The essentially pellagrous area in the United States extends from Maryland to Texas along the coast, and inland to Tennessee, Kentucky, Arkansas and Oklahoma. Some pellagra for like reasons is found in California. A few cases are found in the New England States for the same reasons, also a few in Northern States covered by the glacial drift.

The South Atlantic States are divided into three physiographic regions, the Coastal Plain, the Piedmont Plateau, and the Appalachian Highland. In the Gulf States the Coastal Plain is called the Gulf Coastal Plain. In Alabama the Piedmont Plateau is called the Ashland Plateau. In Missouri, Arkansas and Oklahoma the mountains are called the Ozark, Boston, Oachita, Arbuckle and Wichita mountains. A pre-Cambrian area of highland extends into Texas.

At the western border of the Atlantic Coastal Plain is a distinct scarp marking the commencement of the rocks of the Piedmont Plateau. This is called the "Fall Line." Here all the rivers have falls or rapids as they pass from the granite of the plateau to the sands and clays of the Coastal Plain. Commencing at the south, the Fall Line is found at Columbus, Georgia, extends across Georgia to Augusta, thence northeast across South and North Carolina and Virginia, crossing the James at Richmond and the Potomac at Georgetown. In Texas the Fall Line is called the Balcones Scarp. The soils of the Piedmont and Ashland Plateaus, and of the higher lands of southern Missouri, Arkansas, Oklahoma and Texas are residual clays in place formed from the weathering and disintegration of the igneous and crystalline rocks.

The soils of the Coastal Plains are sedimentary clays washed from the plateau regions. In one region this coastal plain reaches far to the north. This is known geologically as the Mississippi Embayment and extends to the mouth of the Ohio River.

On certain inland slopes of the Appalachian and Ozark highlands there is an extensive deposit of shales of the Mississippian and Pennsylvanian series of the Carboniferous. These shales are highly silicious and weather into clays low in lime. Where the corresponding strata are low in lime there is some pellagra. This condition applies to certain areas in Tennessee, Kentucky, Missouri and Kansas. Nine-tenths of the pellagra in Kentucky is found in the southeastern counties of the State. By far the greater part of pellagra in Kansas is found in the southeastern county, where all the soil is formed from the Cherokee shales.

Certain regions in Illinois, Michigan and Wisconsin as well as northern Missouri are overlaid with a blanket of sand, clay and pebbles called the "glacial drift." This was derived in geologic time from the great pre-Cambrian shield of granite surrounding Hudson's Bay. The original strata of these States is limestone. Where these limestones are covered with the glacial drift to a sufficient thickness to be the source of surface water supplies, there are a few cases of pellagra. The occurrence of pellagra in these regions is limited by the large amount of limestone worked up into the glacial drift.

There are three areas of limestone prairies in the pellagrous regions of the South.

The Cretaceous area marked by the Selma chalk in Mississippi and Alabama.

The Cretaceous area called the "Black Prairies" of Texas.

The Tertiary area underlaid by the Vicksburg and Jackson limestones in Mississippi.

These regions are calcareous and furnish a hard or limestone water. They would naturally be immune as far as pellagra is concerned. However, in geologic time these regions were again submerged and overlaid to a considerable thickness by a sea wash of clay, sand and pebbles. There are two blanket formations of this character. One, the Lafayette formation, extends north to the hills, and where deep enough to be the source of springs and shallow wells, produces freestone water and pellagra even in the limestone areas. The other is the Grand Gulf formation. It covers part of the Jackson formation.

The clay of these areas goes into solution in the water in the form of colloidal silica. Colloidal silica is readily absorbed by the organism. It acts like an enzyme, fixes the salts in the tissues and sets the acids free. At the same time it substitutes a molecule of water. The result of this action is the drying of the tissues so prominent in pellagra, and an acidosis which gives rise to the classical symptoms of chronic acid intoxication which characterize pellagra wherever found in the world.

The cutaneous lesions are characteristic of the acidosis. They are worse in the spring because of the heat. They appear upon the exposed surfaces below the wrist band and above the collar, because these surfaces are exposed to air and sunlight, producing a concentration of the acid by evaporation and an increase of its activity by heat.

The digestive disturbances are typical of an acidosis. To any physician trained in drug and poison indications, the average pellagrin is the victim of chronic poisoning with hydrochloric acid.

The nervous disturbances and the condition of the pleural cavities are the result of the drying up of the fluids occasioned by the substitution of the colloidal silica for water. In colloidal chemistry this is called deaquification because the water is not dried up but is substituted in the chemical combination in the tissues. This is just the same as when tissue is dried and hardened by alcohol. Hence the nervous manifestations of pellagra are analogous to alcoholic intoxication. They are both accomplished by a substitution of water. There is first hyperesthesia, then narcosis, inco-ordination and paralysis.

Since the chlorids predominate in ingestion and in the fluids, the acid most commonly set free by the silica is hydrochloric. If water containing chlorids or sulphates, or materials containing phosphates be ingested, the acids of these salts will also be set free to increase the intoxication. So a water hard with the chlorids or sulphates of calcium will make a pellagrous water worse.

The symptomatology of pellagra is well known to the physicians of the South. It is at once a chronic acidosis and an intoxication due to colloidal silica. The result is the same. One direct, the other secondary. The one thing to remember is that the condition is a chronic acidosis. The prevailing acid is hydrochloric. No theorizing can get away from this fact. That this condition can be produced by the ingestion of colloidal silica there can be no doubt. All the laboratory and domestic animals as well as man are susceptible to the intoxication. Rabbits and guinea pigs, dogs and monkeys, have typical pellagra if fed silica in their food or if given a pellagrogenic water to drink.

Now mineral poisons generally have an antidote. The antidote ta acids is an alkali. The antidotes to the acids which cause the condition called pellagra are the mineral carbonates and the carbonates of the alkaline earths. It is well known that silica is precipitated and rendered inert by the carbonates of sodium, potassium, calcium and magnesium. Because of valence and common accessibility, the carbonate of calcium is the common antidote of nature. It is found in all hard or limestone water. Therefore there is no pellagra in limestone regions. People who drink limestone water do not have pellagra. The limestone regions are prairie. There is no pellagra in distinctly prairie regions.

The prevention of pellagra is a very simple and practical matter. Springs and wells in clay regions should be cleaned out and walled with a good quality of limestone laid in Portland cement mortar. Broken limestone should be placed in the bottom at least a foot deep. The well or spring should be well housed so that clay cannot wash in from the top. The water can be treated from time to time with a little lime. It is only necessary that it be kept at a calcium carbonate content of over 1 per cent. Where these precautions are not practical, draw a bucketful of water and treat it with a little lime. Stir in the lime and permit the water to settle before using it for drinking.

In the pellagrous area city reservoirs should be kept clean of clay. They should be walled with concrete made of good limestone and Portland cement. The bottom should be covered to a foot or two with broken limestone. Part of the filter sand should be limestone screenings. Lime should be used liberally as a coagulant.

Now these precautions are very simple. They are not so profound as to require their submission to the Rockefeller Commission or the Carnegie Foundation. The colored women of the South who do washing and scrubbing know when the water is hard or soft. Abstruse and impossible theorizing about diet, proteids, climate and work does not avail in the presence of such a condition. The insinuation that the people of the South are underfed is a gratuitous insult. It is surprising that such a theory ever found any acceptance in the best fed district of our country. There is no scientific foundation for such a theory. The attempted proof by a nine months diet resulting in scurvy is a modern enormity perpetrated in the name of science. People in pellagrous areas contract pellagra under every and all conditions of domicile, diet, labor and sanitary environment. Under the proper treatment they recover without change of domicile, diet, labor or sanitary environment. These statements are not hypotheses or theories. They are facts well known to all who have studied the subject impartially and without the bias of preconceived hypotheses.

But of what value are hypotheses in the presence of facts? Pellagra is cured anywhere in the world by the administration of the proper alkalinity. This is not disputed. It is confirmed by clinical experience all over the world. Alessandrini and Scala used hypodermic injections of one cubic centimeter of a 10 per cent solution of neutral sodium citrate. They gave the injections once a day at first, then at a longer interval. All their patients recovered in from thirty to sixty days. In a late paper they report similar success all over Italy. The same is true in the United States. A physician in Memphis gave sodium carbonate by the mouth. His patients recovered. Another physician in North Carolina reports uniform success with alkaline treatment. All over the South the same story is told. Persons from the South come North into the limestone areas and bring their pellagra with them. Their skin lesions clear up in thirty days from drinking hard water alone.

The rational treatment of pellagra follows the classical lines of the treatment of any mineral intoxication.

1. Antidote the poison.
2. Eliminate the poisonous products.
3. Build up the depleted organism.
4. Treat the manifestations symptomatically.

The antidotes are the alkaline carbonates and the carbonates of the alkaline earths. Organic alkalies are converted into carbonates in the system, hence sodium citrate is an excellent antidote. Sodium carbonate, calcium carbonate and magnesium carbonate are excellent. Alkalies can be given in the form of milk of magnesia, liquor calcis, calcium lactis and hard or limestone water. Milk of magnesia and calcium lactis will be found excellent to control pyrosis. They can be given in ordinary doses until a desire for acids evidences the control of the acid condition. The bowels must be kept open and the kidneys at their best to eliminate the accumulated mineral solids. There should be no drastic or routine treatment. The whole field of therapeutic materia medica is open to the trained prescriber. After antidoting the poison, prescribe for the patient, not for the pellagra.

Obstinate constipation demands relief by enema first. Do not irritate by laxatives and purgatives. Restore the proper alkalinity and the functions will soon adjust themselves. The skin lesions can be dressed by any lotion which will relieve. The restored alkalinity will soon remove the burning. Mental and nervous disturbances will soon be relieved by the return of the substituted water to the tissues. Hyoscyamus or cannabis indica, or other remedies may be indicated for the peculiar symptomatology of these conditions which simulate an alcoholic intoxication.

To review briefly:

1. Pellagra is a chronic acid intoxication caused by colloidal silica in drinking water.

2. Pellagra is strictly localized and contracted only in those regions where the water commonly used for drinking is derived from clay soils.

3. Pellagra is essentially a disease of a fixed agricultural population living on an exhausted soil of determinate geologic origin.

4. The occurrence of pellagra in the southern part of the United States is merely a coincidence of geology and latitude.

5. The specific origin of pellagra has no relation to domicile, diet, labor or sanitary environment.

6. Any kind of depletion may predispose to pellagra by reducing the resistance and powers of elimination.

7. The poison of pellagra is antidoted by the alkaline carbonates and by the carbonates of the alkaline earths.

8. Pellagra is absolutely prevented by the drinking of water containing a sufficient quantity of the bicarbonates of calcium and magnesium.

9. Pellagra is cured by the simple means of administering a simple and suitable alkaline carbonate or an organic alkali convertible into a carbonate.

10. The manifestations demand symptomatic treatment in addition to the antidote.

[The decision regarding Dr. Lowery's paper is also made out of respect to the paper of Dr. Perdue of Kansas City. In this paper the cause of pellagra is stated to be from an acid condition due to too much silica and too little lime or other alkali. "Sand in his craw" can no longer be popular, although the patients with the most "sand" will continue to improve most rapidly.

Dr. Perdue's paper shows great geological knowledge, but geological conditions are old and pellagra is new. His theory does not explain why a disease could be a scourge in Italy for over two hundred years without being known in America. Nor why when once brought to America it should spread from Panama and the Carolinas to New Mexico in as progressive annual invasions as the cotton boll weevil spread from Mexico through Texas to Mississipi. Something alive there.—Editor.]

